# Maintenance rituximab in Veterans with follicular lymphoma

**DOI:** 10.1002/cam4.3420

**Published:** 2020-08-28

**Authors:** Ahmad S. Halwani, Kelli M. Rasmussen, Vikas Patil, Deborah Morreall, Catherine Li, Christina Yong, Zachary Burningham, Keith Dawson, Anthony Masaquel, Kevin Henderson, Elisha DeLong‐Sieg, Brian C. Sauer

**Affiliations:** ^1^ Division of Hematology and Hematologic Malignancies Huntsman Cancer Institute Salt Lake City Utah USA; ^2^ Division of Epidemiology VERITAS University of Utah Salt Lake City Utah USA; ^3^ VERITAS George E Wahlen Department of Veterans Affairs Medical Center Salt Lake City Utah USA; ^4^ US Medical Affairs Genentech Inc South San Francisco CA USA

**Keywords:** clinical observations, epidemiology, haematological cancer, lymphoma

## Abstract

Real‐world practice patterns and clinical outcomes in patients with follicular lymphoma (FL), including the adoption of maintenance rituximab (MR) therapy in the United States (US), have been reported in few studies since the release of the National LymphoCare Study almost a decade ago. We analyzed data from the largest integrated healthcare system in the United States, the Veterans Health Administration (VHA), to identify rates of adoption and effectiveness of MR in FL patients after first‐line (1L) treatment. We identified previously untreated patients with FL in the VHA between 2006 and 2014 who achieved at least stable disease after chemoimmunotherapy or immunotherapy. Among these patients, those who initiated MR within 238 days of 1L composed the MR group, whereas those who did not were classified as the non‐MR group. We examined the effect of MR on progression‐free survival (PFS) and overall survival (OS). A total of 676 patients met our inclusion criteria, of whom 300 received MR. MR was associated with significant PFS (hazard ratio [HR]=0.55, *P* < .001) and OS (HR = 0.53, *P* = .005) compared to the non‐MR group, after adjusting by age, sex, ethnicity, geographic region, diagnosis period, stage, grade at diagnosis, hemoglobin, lactate dehydrogenase (LDH), Charlson comorbidity index (CCI), 1L treatment regimen, and response to 1L treatment. These results suggest that in FL patients who do not experience disease progression after 1L treatment in real‐world settings, MR is associated with a significant improvement in both PFS and OS. Maintenance therapy should be considered in FL patients who successfully complete and respond to 1L therapy.

## Graphical abstract

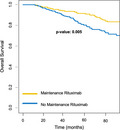

## 1 INTRODUCTION

Follicular lymphoma (FL) is the most common indolent non‐Hodgkin lymphoma.^1^ While most patients are diagnosed with advanced disease, a majority have an excellent prognosis with a disease trajectory that can span decades, despite the fact that treatment options often carry substantial morbidity and are ultimately not curative.^2^, ^3^, ^4^ Many FL patients present with aggressive disease with short term responses, frequent relapses, and early mortality.^5^, ^6^ Given the heterogeneity in FL’s disease trajectory, treatment selection must carefully balance efficacy and toxicity.^7^, ^8^

There remains no one particular standard of care for first‐line (1L) treatment in patients with FL,^9^ and no consensus on how to appropriately adapt 1L treatment based on individual patient and disease characteristics.^8^ Patients are commonly treated with a combination of chemotherapy and immunotherapy, with the option to be followed by observation or receive “maintenance therapy” following the successful completion of 1L treatment. Often, maintenance therapy consists of the monoclonal antibody used during 1L treatment.^9^ Available evidence examining the benefit of maintenance therapy is mixed, with no consensus as to whether maintenance therapy improves overall survival (OS), or which maintenance therapy is superior. As a result, maintenance therapy remains controversial.^10^

Real‐world evidence is increasingly recognized as an important complement to randomized clinical trial (RCT)‐derived evidence.^11^, ^12^, ^13^ Using data from insurance claims or electronic healthcare records, real‐world evidence provides information about the treatment options that are adopted by patients and physicians outside of a clinical trial setting. Real‐world evidence is particularly important in those patient populations that are often under‐represented in RCTs, such as patients who are typically older, carry higher comorbidity burdens, or are more racially/ethnically diverse. In an effort to advance our understanding of the use of maintenance therapy in FL patients, we conducted a real‐world study examining the treatment practices and outcomes in Veterans with FL, specifically the use of maintenance therapy after the successful completion of 1L treatment.

## 2 METHODS

### 2.1 Cohort definition; patient and disease characteristics; treatment practices

We used Veterans Affairs Cancer Registry System (VACRS)^14^, ^15^ data as of March 2017 to identify patients diagnosed with FL (ICD‐O‐3 codes 96903, 96953, 96913, or 96983) in the largest integrated healthcare system in the United States, the Veterans Health Administration (VHA), from January 2006 to December 2014. Patients without a hematology/oncology visit within 6 months of the diagnosis date were excluded as these were likely patients who were diagnosed and treated outside the VHA and whose healthcare management we therefore had limited ability to observe. Patients with a VACRS record of another malignancy prior to the diagnosis of FL were also excluded. The resulting patients were then followed until end of study observation period (December 2016), absence of hematology/oncology services utilization for more than 18 months, a nonlymphoma malignancy, or death. Since most bendamustine utilization occurred in or after 2010, patients were divided into early (2006–2009) and late (2010–2014) cohorts according to diagnosis date.

We extracted date of birth, sex, race/ethnicity, and residence at diagnosis from the VHA Corporate Data Warehouse (CDW). Patients' residential ZIP code was used to identify geographic region of residence in accordance with the defined regions of the US Census Bureau. FL grade and stage at diagnosis were extracted from VACRS, or, when necessary, from pathology and clinical notes. We did not collect data on the proportion of patients meeting Groupe d'Etude des Lymphomes Folliculaires^16^ criteria for high tumor burden because patients in the VHA who do not meet treatment criteria (including high tumor burden) simply do not receive treatment; since all patients included in the study received treatment, we assume that all patients in the study had high tumor burden.

In order to study outcomes associated with adoption of MR after immunotherapy or chemoimmunotherapy in patients with nonlocalized disease, we defined a target study population of “MR‐eligible” patients who were diagnosed with FL stage II–IV and grade 1–3a; received an anti‐lymphoma immunotherapy or chemoimmunotherapy for at least 21 days; achieved complete, partial, or stable response after the completion of 1L treatment; and were either observed or initiated on MR within 238 days of 1L—the period of time in which 95% of MR patients were initiated on their maintenance treatment and which was prespecified prior to the final analysis. We included patients with stable disease after 1L because we are aware that some clinicians prescribe MR in patients with stable response after 1L, and we sought to include as representative a population of real‐world patients as possible.

To extract and classify treatment regimens, we identified chemotherapy agents typically used in FL treatment (Table S1) by review of National Comprehensive Cancer Network (NCCN) B‐Cell Lymphoma guidelines (NCCN, 2017). We retrieved single‐agent dispensation information from the CDW and used a rule‐based algorithm to classify co‐administration of multiple agents into NCCN‐concordant lines. Most FL patients identified received 1 of 4 1L treatment regimens—RCHOP (rituximab combined with cyclophosphamide, doxorubicin, vincristine, and prednisone), RCVP (rituximab combined with cyclophosphamide, vincristine, and prednisone), BR (bendamustine and rituximab), or single‐agent rituximab. Therefore, our study examined the treatment practices and outcomes of FL patients who received RCHOP, RCVP, BR, or single‐agent rituximab as their 1L treatment. These patients then underwent a manual chart review by an experienced research data coordinator to confirm diagnosis, treatment, and response assessment. Patients with incomplete clinical documentation were considered nonevaluable and were excluded from the final analysis (n = 87).

In addition, we extracted FL International Prognostic Index (FLIPI)^4^ risk factors available in CDW, namely hemoglobin and lactate dehydrogenase (LDH). We also used Quan's algorithm to calculate Charlson comorbidity index (CCI) based on ICD‐9‐CM and ICD‐10 codes in inpatient and outpatient visits within a 1‐year lookback prior to treatment initiation.^17^, ^18^ Vital status and date of death for patients who died during the study were obtained from CDW, which aggregates vital status from multiple sources.

### 2.2 Statistical analysis

Descriptive statistics were used to describe patient demographics, disease characteristics, and treatment practice patterns. Proportions were used for discrete variables; means and/or medians (with standard deviation or interquartile range, respectively) were used to describe continuous variables. Chi‐square test was used to compare categorical variables between different treatment groups. Age and CCI groups were defined based on how these variables are dichotomized in prognostic indices, namely FLIPI^4^ and FLIPI/CCI/histological grade,^19^ respectively. Missing variables (ethnicity, region, stage, grade, hemoglobin, and LDH) were imputed using a random forest‐based model^20^ that included age, sex, ethnicity, CCI at diagnosis and before 1L, region, year of diagnosis, stage, grade, hemoglobin, LDH, and 1L treatment.

Patients were censored at documentation of another malignancy in VACRS, or end of study observation period. For treatment history reconstruction, patients were also censored at the day of last hematology/oncology visit or anticancer treatment dispensation if, subsequently, 18 months lapsed without evidence of either in the medical record.

Diligent effort was expended to identify and address potential sources of bias. In particular, we prospectively identified several sources of possible bias, including information bias related to variable extraction; confounding by indication due to an imbalance in baseline patient and disease characteristics, type of 1L treatment, and the responses achieved following the completion of 1L treatment; and, lastly, immortal time bias,^21^, ^22^, ^23^ which arises due to the fact that patients who initiate MR are essentially ‘immortal’ from the time they conclude 1L treatment to the time MR is initiated, granting them an unfair survival advantage over patients who are not initiated on MR after 1L. To avoid these biases, we systematically extracted and harmonized information from multiple data sources in the CDW, then completed a thorough chart review by a human annotator to ensure that patient diagnoses, treatments, and responses were accurately extracted. Additionally, in order to further avoid immortal time bias, we used a landmark analysis.^24^ For the purposes of this study, the landmark period started at the completion of 1L treatment and ended at the landmark threshold of 238 days. This landmark threshold was determined prior to executing the final analysis so that approximately 95% of the patients who received MR were classified in the MR group. This threshold is similar to thresholds chosen by prior studies which examined the distribution of the number of days between the completion of 1L treatment and the initiation of MR.^25^ Progression‐free survival (PFS) and OS were defined as time from the end of the landmark period to progression or death, respectively. To address confounding by indication, we then used a Cox proportional hazards model^26^ to compare PFS and OS while adjusting for patient demographics, available baseline FL risk factors, diagnosis period, 1L treatment received, and 1L treatment response achieved prior to initiation of MR or observation.

Sensitivity analyses were performed to assess the effects of varying the length of the landmark threshold period (Table S3) on the study's conclusions.

## 3 RESULTS

### 3.1 Patient Demographics, disease characteristics, and 1L treatment practices

**Figure 1:**
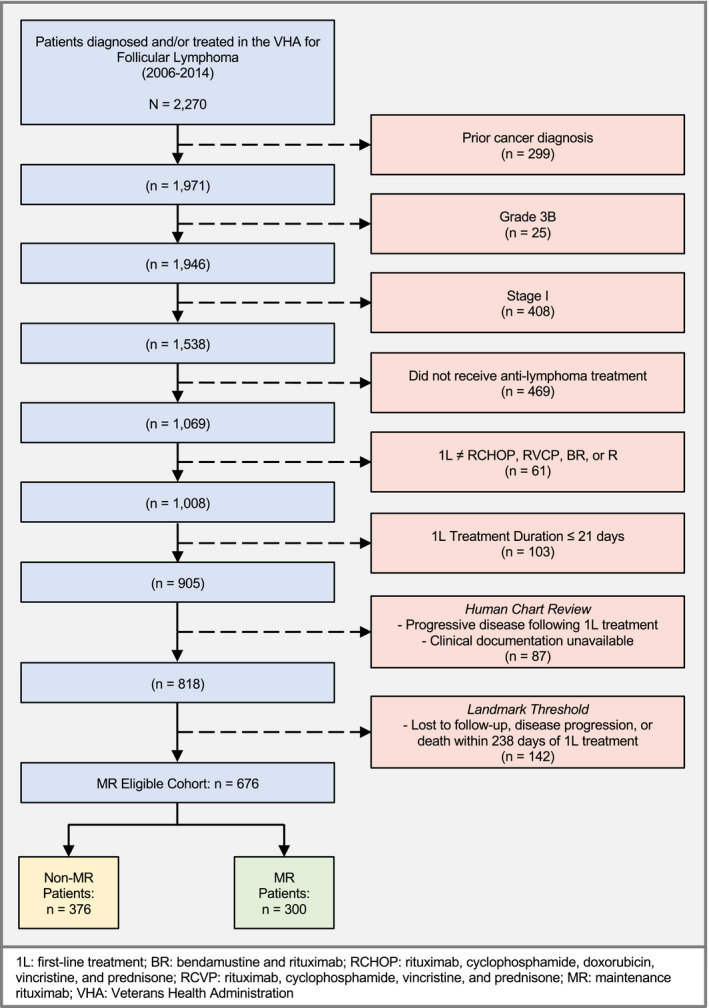
Cohort Attrition Figure

From 2006 to 2014, 2,270 patients were diagnosed with FL at the VHA and made up the FL cohort (Table S2 compares characteristics of the FL cohort to those reported by NLCS and Surveillance, Epidemiology, and End Results Program [SEER]).^27^, ^28^ After excluding patients with prior cancer or documented grade 3b or stage I disease; patients without documented anticancer treatment in the VHA based on pharmacy dispensation records; and patients whose dispensation records were incompatible with administration of RCHOP, BR, RCVP, or single‐agent rituximab for at least 21 days, 905 patients remained (Figure 1).

After human chart review, 59 of these patients were excluded due to lack of retrievable clinical documentation regarding treatment events (25 patients) or responses after 1L (34). Of the remaining patients, 28 had progressive disease after 1L treatment and were therefore considered not MR‐eligible. Finally, 676 patients were followed up for at least 238 days with no sign of progression—the MR‐eligible cohort comprised these patients, of whom 300 received MR (44%) and 376 (56%) did not (Figure 1).

Median age was 63; 95% of patients were male; and 82% were non‐Hispanic white, 7% were black, 2% were Hispanic, and the remaining 9% were “other” or unknown race. Approximately 25% of patients resided in the South, 20% the Midwest, 16% the West, and 12% the Northeast (Figure S1). Region of residence was not available for 28% of patients.

Prevalence of FL risk factors in MR‐eligible patients was as follows: 444 (66%) were age >60, 129 (19%) had high‐grade (grade 3 or grade 3a) disease, 526 (78%) had stage III or stage IV at diagnosis, 156 (23%) had a hemoglobin <12 g/dl prior to initiation of 1L, and 125 (18%) had LDH > upper limit of normal (ULN) prior to initiation of 1L. Median CCI at treatment was 2 (interquartile range [IQR] 0–3) (Table 1). Hemoglobin and LDH were not available in 8 (1%) and 82 (12%) of patients, respectively.

**Table 1:** Patient Demographics, Treatment Patterns, and Baseline Disease Characteristics, 2004‐2016

	MR^†^‐Eligible	MR	Non‐MR	*P*‐value
Characteristic, n (%)	N = 676	n = 300	n = 376	
Age > 60	444 (66)	199 (66)	245 (65)	.81
Sex, male	641 (95)	286 (95)	355 (94)	.72
Ethnicity				.44
White	554 (82)	245 (82)	309 (82)	
Black	48 (7)	19 (6)	29 (8)	
Hispanic	16 (2)	6 (2)	10 (3)	
Other	22 (3)	13 (4)	9 (2)	
Unknown	36 (5)	17 (6)	19 (5)	
Geographic Region				.20
Midwest	132 (20)	59 (20)	73 (19)	
Northeast	78 (12)	39 (13)	39 (10)	
South	172 (25)	81 (27)	91 (24)	
West	106 (16)	38 (13)	68 (18)	
Unknown	188 (28)	83 (28)	105 (28)	
CCI at 1L				.30
0–1	321 (48)	150 (50)	171 (46)	
2–14	353 (52)	150 (50)	203 (54)	
Histology, Grade				.07
1	170 (25)	84 (28)	86 (23)	
1–2	38 (6)	17 (6)	21 (6)	
2	191 (28)	85 (28)	106 (28)	
3	88 (13)	29 (10)	59 (16)	
3a	41 (6)	13 (4)	28 ( 7)	
Unknown	148 (22)	72 (24)	76 (20)	
Stage				.50
II	108 (16)	53 (18)	55 (15)	
III	285 (42)	122 (41)	163 (43)	
IV	241 (35)	111 (37)	130 (35)	
Unknown	42 (6)	14 (5)	28 (7)	
Hemoglobin < 12 g/dL	156 (23)	56 (19)	100 (27)	.02
LDH > ULN	125 (18)	56 (19)	69 (18)	.95
1L Treatment Regimen				.37
RCHOP	243 (36)	106 (35)	137 (36)	
RCVP	190 (28)	78 (26)	112 (30)	
BR	159 (24)	72 (34)	87 (23)	
R	84 (12)	44 (15)	40 (11)	
Diagnosis period				.18
2006–2009	293 (43)	121 (40)	172 (46)	
2010–2014	383 (57)	179 (60)	204 (54)	
1L Treatment Response				<.001
CR	336 (50)	116 (39)	220 (59)	
PR	315 (47)	180 (60)	135 (35)	
SD/No Response	21 (3)	*	20 (5)	
Unknown	*	*	*	
Response after MR			N/A	N/A
CR	196 (29)	196 (65)		
PR	85 (13)	85 (28)		
SD	13 (2)	13 (4)		
Unknown	*	*		

Abbreviations: 1L, first‐line treatment; BR, bendamustine and rituximab; CCI, Charlson comorbidity index; CR, complete response; LDH, lactate dehydrogenase; MR, maintenance rituximab; N/A, not applicable; PR, partial response; R, rituximab; RCHOP, rituximab, cyclophosphamide, doxorubicin, vincristine, and prednisone; RCVP, rituximab, cyclophosphamide, vincristine, and prednisone; SD, stable disease; ULN, upper limit of normal.

* Fewer than five patients were identified.

The most common comorbidity was diabetes, present in 143 (21%) of patients, followed by pulmonary disease (134, 20%), renal disease (68, 10%), and peripheral vascular disease (60, 9%). Median time from diagnosis to 1L initiation was 56 days (IQR 30–105 days). The most commonly received 1L treatments for FL patients were: RCHOP (243, 36%), followed by RCVP (190, 28%), BR (159, 24%), and single‐agent rituximab (84, 12%).

### 3.2 MR adoption

Of 676 MR‐eligible patients, 300 patients (44%) received MR. Patients who received MR differed from those who did not receive MR in that a lower proportion of MR patients met the FLIPI criterion for hemoglobin <12 (19% vs 27%, *P* = .022). Patients who received MR were also less likely to have achieved a complete response following 1L treatment (39% vs 59%, *P* < .001) and were more likely to have achieved a partial response (60% vs 36%, *P* < .001). The two groups were comparable in age, sex, ethnicity, geographical residence, CCI at 1L, grade, stage, proportion of patients with LDH > ULN, and 1L treatment regimen (Table 1). MR adoption did not vary significantly between earlier and later study periods, with 121 of 293 earlier patients (41%) and 179 of 383 later patients (456%) receiving MR (*P* = .18).

Of 300 patients whose MR dispensation schedule was evaluable (Table 2), 114 (38%) received MR every 2 months, 82 (27%) every 3 months, and 75 (25%) weekly for 4 weeks every 6 months. The most common dose frequency changed by diagnosis period. While most patients receiving MR in the earlier period received rituximab weekly for 4 weeks every 6 months (56 out of 121 patients, 46%), the majority of later MR patients received rituximab every 2 months (102 out of 179 patients, 57%). MR was started at a median of 2 months (IQR 2–3 months), 3 months (IQR 3–4 months), and 6 months (IQR 3–6 months) after 1L completion in patients receiving MR every 2 months, 3 months, and weekly for 4 weeks every 6 months, respectively. Median duration of treatment for patients receiving rituximab every 2 months and 3 months was 21 months (IQR 13–22 months) and 20 months (IQR 17–23 months), respectively. The median duration of treatment for those receiving rituximab weekly for 4 weeks every 6 months was 15 months (IQR 7–20 months).

**Table 2:** MR Dose Number, Frequency, and Duration of Treatment

MR Frequency	All Patients (N = 300)					Earlier Epoch (n = 121)		Later Epoch (n = 179)	
	n	%	Time to Start MR,^a^ median (IQR)	Number of MR Doses,^b^ median (IQR)	Duration of Treatment,^c^ median (IQR)	n	%	n	%
q2 months	114	38%	2 months (2–3)	12 (8–12)	21 months (13–22)	12	10%	102	57%
q3 months	82	27%	3 months (3–4)	8 (6.25–9)	20 months (17–23)	38	31%	44	25%
q6 months (4 weekly)	75	25%	6 months (3–6)	13 (8–16)	15 months (7–20)	56	46%	19	11%
other	29	10%	–	–	–	15	12%	14	8%

Note

IQR, interquartile range; MR, maintenance rituximab; q, quarter.

^a^ Time from last dispensation of first‐line treatment to first dispensation of MR in months.

^b^ Doses given on the same week were counted once only.

^c^ Time from first dispensation of MR to last dispensation of MR in months.

### 3.3 Clinical outcomes: response rates

Of the 300 patients who were treated with MR, 116 (39%) and 180 (60%) had achieved a CR and PR, respectively, prior to MR initiation. The low number of patients with stable disease prior to MR initiation provided insufficient power to comment on survival rates in this specific population in analyses below. After MR, 196 (65%) achieved a complete response, 85 (28%) achieved a partial response, 13 patients (4%) had stable disease, and fewer than six patients (<2%) had an undetermined response following MR.

### 3.4 Clinical outcomes: progression‐free survival

**Figure 2:**
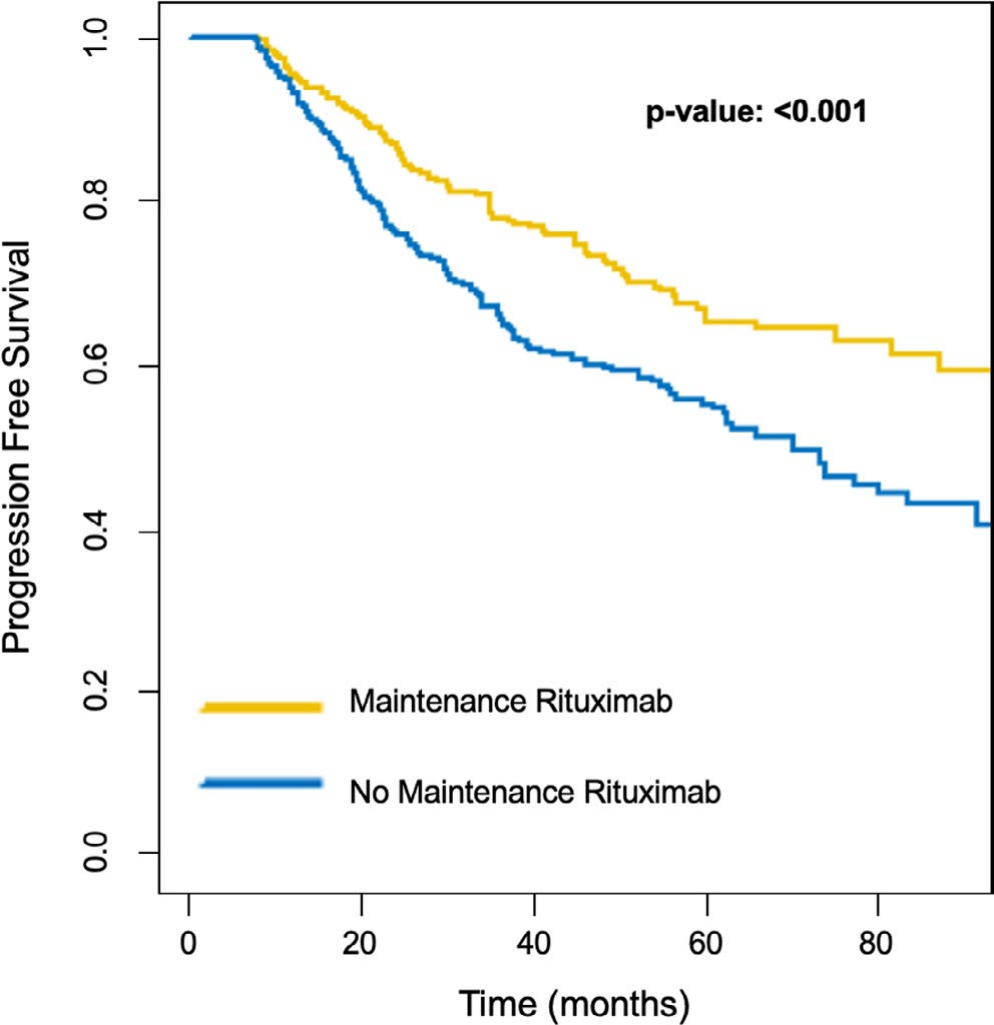
Progression‐Free Survival, unadjusted Kaplan‐Meier Curves

During a median follow‐up of 3.75 years (IQR 2.04–5.88 years) from landmark time, The MR group experienced 93 progression events, whereas the non‐MR group underwent 144 events. Median PFS of patients receiving MR was 8.83 years from landmark time, as opposed to 5.79 years from landmark time for non‐MR patients. Figure 2 shows the unadjusted Kaplan‐Meier survival curves for progression free survival stratified by MR and non‐MR groups.

In a multivariable Cox model stratified by 1L treatment and adjusted for patient characteristics including age, sex, race/ethnicity, region, CCI, disease risk factors (stage and grade at diagnosis, hemoglobin, LDH), response achieved prior to MR initiation, and diagnosis period; MR was associated with an increase in PFS (hazard ratio [HR]=0.55, *P* < .001). In the same analysis, stage IV disease (HR = 2.10, *P* < .001) and partial response after 1L (HR = 1.7, *P* < .001) were independently associated with a shorter PFS.

### 3.5 Clinical outcomes: overall survival

**Figure 3:**
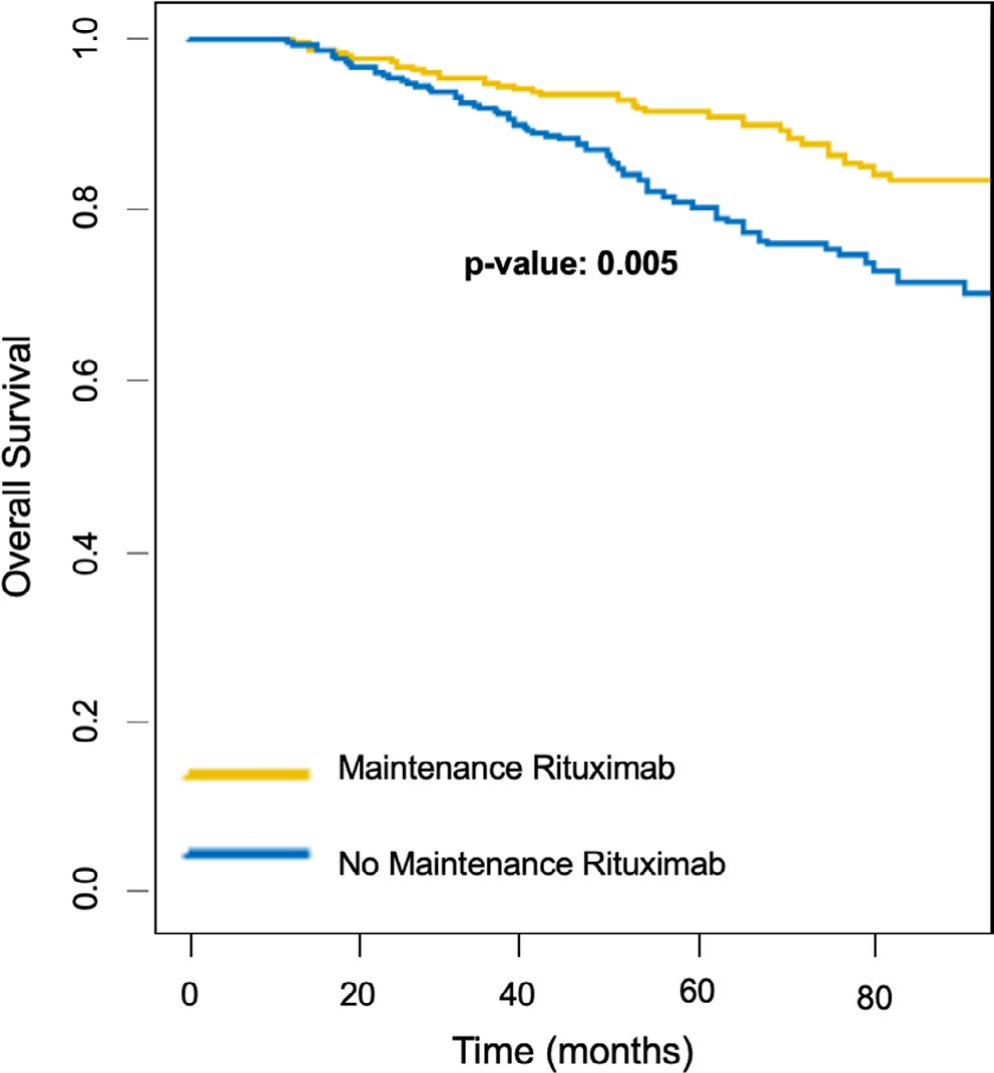
Overall Survival, unadjusted Kaplan‐Meier Curves

The unadjusted Kaplan‐Meier survival curves for OS stratified by MR and non‐MR groups are shown in Figure 3. MR was also associated with a prolonged OS compared to the non‐MR group. The MR group had 35 deaths compared to 73 deaths in the non‐MR group. Both the median OS for the non‐MR group and the MR group were not reached in an unadjusted analysis.

In a multivariable Cox model stratified by 1L treatment and adjusted for the same covariates described above, including patient and disease characteristics, response achieved prior to MR initiation, as well as diagnosis period; MR was associated with an increase in OS (HR = 0.53, *P* = .005). In the same analysis, age > 60 (HR = 1.9, *P* = .006), partial response after 1L (HR = 2.3, *P* < .001), and stable/no response after 1L (HR = 4.5, *P* < .001) were independently associated with shorter OS, whereas later diagnosis was associated with longer OS (HR = 0.52, *P* = .03). MR was associated with OS even with slightly shorter landmark durations of 211 days and 187 days, where 90% and 85% of MR initiations fell within the landmark period, respectively (Table S3).

## 4 DISCUSSION

Despite FL's prevalence as the most common indolent lymphoma,^29^, ^30^, ^31^ there is no standard of care for 1L treatment.^9^ Commonly used regimens over the last decade include an immunotherapy agent (traditionally single‐agent rituximab, or more recently obinutuzumab, a second‐generation Type II monoclonal antibody) combined with a chemotherapy backbone such as CHOP, CVP, or bendamustine. Evidence about their comparative efficacy comes largely from clinical trials, which have failed to demonstrate a consistent OS advantage of one regimen over another.^2^, ^32^, ^33^, ^34^, ^35^, ^36^, ^37^, ^38^ Older patients and those with low tumor burden and/or comorbidities are often treated with single‐agent rituximab.^39^, ^40^, ^41^, ^42^, ^43^ Patients are then either observed or offered maintenance treatment based on evidence from a series of clinical trials that demonstrated improvement in PFS but not necessarily OS.^40^, ^44^, ^45^, ^46^ In a systematic review and meta‐analysis of RCTs, MR was associated with improved OS, primarily in the relapsed or refractory setting.^47^

Several reports have recently described real‐world 1L practices and outcomes in FL in relation to the use of MR.^48^, ^49^, ^50^, ^51^ For instance, a German study reported improvement in complete response rates with maintenance therapy,^48^ whereas Nordic^49^ and Czech^50^, ^51^ studies reported improvement in OS with MR. In contrast, the largest—and, to date, only—nationwide study of real‐world FL practices and outcomes in the United States, the NLCS,^27^ did not reveal an association between MR and OS.^52^ NLCS reported improvements in PFS and time to next treatment with use of MR, but no improvement in OS.

Our objective was to describe adoption of MR in a nationwide cohort of patients and the clinical outcomes (disease response, PFS, and OS) associated with this practice, by examining treatment practices and outcomes in the largest integrated health system in the United States, the VHA. In order to accomplish this, we also had to determine how representative these Veteran patients were of FL patients in the United States.

To the best of our knowledge, the distribution of demographic and disease‐specific characteristics of FL patients treated in the VHA has not been previously reported. With the exception of gender, we found that the distributions of demographic and disease‐specific characteristics of the FL Veteran patient population were similar to those reported in real‐world studies of FL including SEER and NLCS,^27^, ^28^ with the rates of missing data observed in our study comparable to those observed in the same prospective registries (Table S2). In addition, the characteristics of the MR‐eligible patients in our study were similar to those of patients described in the Nordic collaborative (NC)^49^ and the Czech Lymphoma Study Group (CLSG) studies.^50^ These studies reported an average patient age of 61 years (NLCS, NC) to 70 years (CLSG), 76–87% with stage III/IV disease, and 19%–24% with grade 3a disease (NC).

At the initiation of our study, we anticipated two major shifts in treatment practices of previously untreated patients with FL: the increasing adoption of BR after bendamustine's FDA approval in 2008 and increasing adoption of MR as results from studies such as PRIMA became more available to the practicing community.^44^ Both practices were supported by studies that had reported an improvement in PFS, but not OS in patients receiving BR in 1L and MR, respectively. Our results demonstrate a brisk adoption of BR and of PRIMA’s schedule for MR, when the latter is administered. Our cohort showed almost no utilization of 1L BR prior to 2010, yet BR had become the most common 1L treatment in patients diagnosed in 2014. The increased adoption of BR for 1L treatment in FL patients treated in the United States has also been reported by other studies examining 1L treatment practices patterns.^53^ For the majority of patients who received MR in the latter period, the dosing frequency and duration of treatment aligned with that of the PRIMA study.^44^ While BR was quickly adopted as the most common 1L treatment, MR utilization remained stable and a minority of patients received MR after 1L treatment. Following the landmark threshold adjustment, approximately 44% of patients in our cohort received MR, comparable to the rate reported in NLCS (45%) and lower than those reported in NC (50%) and CLSG (68%), indicating a difference in how the evidence regarding MR has been translated to practice by patients and physicians in different countries.^27^, ^49^, ^50^

While our study did not involve a qualitative component to examine attitudes behind this adoption pattern, we believe it likely reflects the controversy regarding the efficacy of MR. In fact, during our human chart review, we found documentation of discussions as to whether to pursue MR, with physicians often relaying to patients that while MR improves PFS, it does not improve OS.

Our study highlights the emerging pattern of increased utilization of MR in patients who achieve partial response. As a result, it is critical that observational real‐world studies of FL patients who receive MR adjust for responses achieved in 1L treatment when comparing outcomes of MR treatment.

Finally, our study joins two other studies examining the use of MR in FL patients treated in real‐world settings which demonstrate an association between MR and OS benefit.^49^, ^50^ It is therefore important for clinicians to inform patients that conflicting evidence exists about the benefits of MR in this setting, rather than simply reporting an absence of OS benefit. The absence of OS benefit in PRIMA may be due to enrollment of a younger and healthier group of FL patients than is typically encountered in real‐world settings.^44^ In addition, the NLCS study found no OS benefit in MR treatment.^25^ Possible factors may include the exclusion of patients who did not complete the entire 1L treatment regimen, factors related to MR dosing (in our cohort, patients receiving MR tended to have a higher frequency and duration of rituximab administration than those treated in the same era as NLCS patients), or factors related to our patient population being overwhelmingly male, and therefore potentially bearing higher‐risk disease than the NLCS cohort.^54^, ^55^

Limitations of this study include restricted follow‐up, especially for patients indexed later in the cohort. In addition, the patient population was drawn entirely from VHA patients, of whom 95% are male, which previous studies have demonstrated to be an independent factor in survival among patients receiving rituximab.^54^, ^55^

## 5 CONCLUSION

The results of this real‐world study suggest that MR after the completion of 1L treatment is not commonly adopted in Veterans with FL, and there has been little to no increase in adoption over time. Patients who achieve a partial response to 1L treatment are more likely to receive MR. Our study joins two other studies of FL in real‐world settings and one meta‐analysis of clinical trial evidence, suggesting that MR is associated with an increase in OS in FL patients. Physicians should incorporate these findings, rather than relying exclusively on the findings of the PRIMA study when recommending whether a patient should receive MR after the completion of 1L treatment. Based on our study, maintenance therapy after 1L treatment in FL should be considered, especially in those patients who may not be as young or fit as those reported in clinical trials.

## CONFLICT OF INTERESTS

AS Halwani has received research grant support from Bristol Myers Squibb, Kyowa Hakko Kirin, Seattle Genetics, Roche, Genentech, Miragen, Immunedesign, Takeda, Amgen, Pharmacyclics, and AbbVie. BC Sauer has received research grant support from Roche, Genentech, Pharmacyclics, and AbbVie. K Dawson, A Masaquel, K Henderson, and E DeLong‐Sieg are employees of Genentech and may own Roche stocks/stock options. KM Rasmussen, V Patil, C Li, D Morreall, C Yong, and Z Burningham certify that they have no declaration of interests.

## AUTHORS’ CONTRIBUTION

The study was sponsored by Genentech, Inc. All authors contributed to the design and objectives of the study. A Halwani, B Sauer, Z Burningham, and A Masaquel were responsible for the study design and methodology. A Halwani and V Patil developed and implemented the R pipelines and modules. The results were interpreted by A Halwani, K Rasmussen, and C Li, whereas the implications for clinical practice were interpreted by A Halwani, K Rasmussen, D Morreall, K Dawson, A Masaquel, K Henderson, and E DeLong‐Sieg. The draft manuscript was prepared by A Halwani and K Rasmussen, with medical writing services provided by Christina Yong on behalf of the University of Utah and the George E Wahlen Veterans Health Administration. It was reviewed, edited, and revised by all authors. Yong Mun, Principal Statistical Scientist for Genentech provided statistical guidance and review of the manuscript.

## Supporting information

Fig S1

Table S1‐S3

## Data Availability

The data that support the findings of this study are available through the Veterans Health Administration. Restrictions apply to the availability of these data, which were approved under the University of Utah Institutional Review Board #03982 and the VA Salt Lake City Human Research Protection Program.
