# Supplementary figures and images for: Maintenance rituximab in Veterans with follicular lymphoma
Source: Cancer Med. 2020 Aug 28;9(20):7537–47. doi: 10.1002/cam4.3420 (PMC7571803; doi:10.1002/cam4.3420)

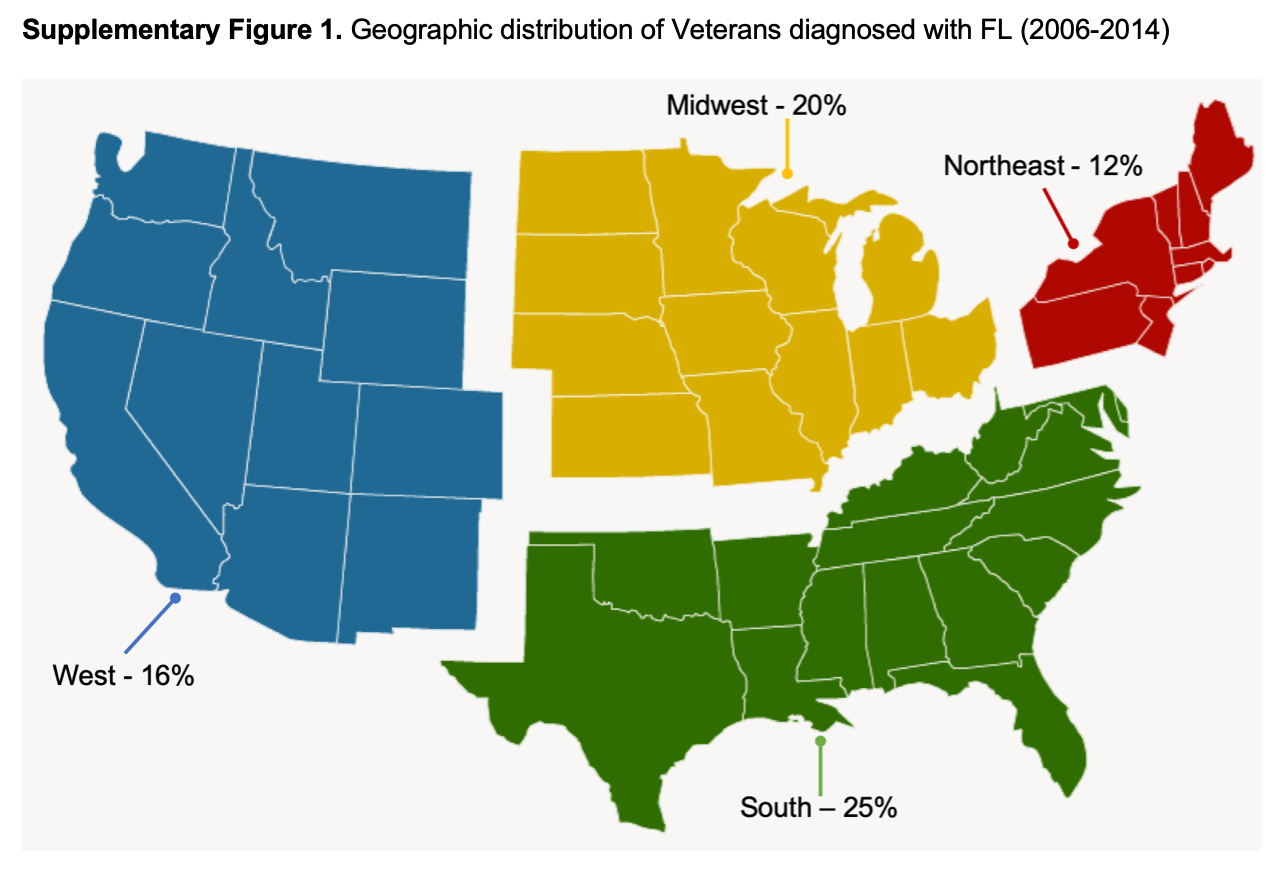

Supplement: Supplementary file 1 — Fig S1 [file CAM4-9-7537-s001.png]
