# Supplementary material for: Maintenance rituximab in Veterans with follicular lymphoma
Source: Cancer Med. 2020 Aug 28;9(20):7537–47. doi: 10.1002/cam4.3420 (PMC7571803; doi:10.1002/cam4.3420)
Supplement: Supplementary file 2 — Table S1‐S3 [file CAM4-9-7537-s002.docx]

# Supplementary Tables

| **Supplementary Table 1.** NCCN Therapeutic Agents for Treatment of B-cell Lymphoma | | | | | |
| --- | --- | --- | --- | --- | --- |
| chlorambucil | oxaliplatin | bendamustine | cladribine | venetoclax | ifosfamide |
| obinutuzumab | cytarabine | fludarabine | cisplatin | lenalidomide | methylprednisolone |
| ibrutinib | mitoxantrone | cyclophosphamide | carboplatin | alemtuzumab | procarbazine |
| ofatumumab | dexamethasone | pentostatin | etoposide | doxorubicin | brentuximab vedotin |
| rituximab | prednisone | idelalisib | gemcitabine | vincristine | mesna |
| NCCN: National Comprehensive Cancer Network | | | | | |

| **Supplementary Table 2.** VHA FL Patient Demographics Compared to NLCS and SEER | | | |
| --- | --- | --- | --- |
|  | **VHA**  **2006–2014** | **NLCS**  **2004–2007** | **SEER 2004** |
| Characteristic | **N = 2,270** | **N = 2,728** | **N = 2,756** |
| Median age, years | 65 | 61 | 63 |
| Sex, female % | 4.3 | 51.8 | 50.6 |
| Ethnicity (%)  White  Black  Hispanic  Other  Unknown | 81.3  7.8  2.2  3.1  5.6 | 90.4  3.4  *  6.1  * | 89.6  4.1  *  6.3  * |
| Histology, Grade, %  1  1-2  2  3  3a  3b  Unknown | 25  5  28  12  5  1  24 | 43  *  29  19  *  *  10 | 27  23  *  19  *  *  33 |
| Stage, %  I  II  III  IV  Unknown | 21  14  30  28  7 | 17  15  29  37  1 | 29  16  22  26  7 |
| FL: Follicular Lymphoma; NLCS: National LymphoCare Study, SEER: Surveillance, Epidemiology, and End Results; VHA: Veterans Health Administration, *Information not available | | | |

| **Supplementary Table 3.** Hazard Ratio by Different Landmark Thresholds for MR | | | |
| --- | --- | --- | --- |
| **Threshold** | **N** | **HR** | **p-value** |
| 0.95 (238 days) | 676 | 0.53 | 0.005 |
| 0.90 (211 days) | 692 | 0.56 | 0.009 |
| 0.85 (187 days) | 706 | 0.52 | 0.004 |
| HR: Hazard Ratio; MR: Maintenance Rituximab; N: Number of Observations after Landmark | | | |
